# 

**Published:** 1917-03-10

**Authors:** 


### Matrons' and Sisters' Appointments.

GILBERT BAIN HOSPITAL, LERWICK. — Miss Margaret Maclean has been appointed matron. She was trained at Leith General Hospital, where she was later night superintendent and assistant matron, and at Edinburgh City Hospital.

HAWKMOOR SANATORIUM, NEAR BOVEY TRACEY.—Miss Ethel Maude Jackson has been appointed matron. She was trained at the Royal Devon and Exeter Hospital, where she was later ward sister, and then assistant matron.

ROYAL ALEXANDRA INFIRMARY, PAISLEY.—Miss Cowie has been appointed matron. She was trained at the infirmary, where for five years she was sister of the *x*-ray and out-patient departments, and has been on the private staff. In 1914 Miss Cowie gained the Florence Nightingale Medal offered by the Scottish Trained Nurses' Association for the best essay on "Modern Nursing."

ROYAL NATIONAL SANATORIUM FOR CONSUMPTION, BOURNEMOUTH.—Miss Edith Ellis has been appointed matron. She was trained at Charing Cross Hospital, where later she held the post of theatre sister. She has also held the following posts: Theatre sister at Wolverhampton General Hospital; sister at the Royal National Hospital for Consumption, Ventnor; acting matron at the Rustington Convalescent Home for Men, Littlehampton; sister (and at times acting matron) of the Royal National Sanatorium, Bournemouth.

MILE END MILITARY HOSPITAL.—Miss Flora C. Griggs has been appointed assistant matron. She was trained at St. Pancras Infirmary, and her subsequent appointments have been ward sister at the Park Hospital, night sister at St. Pancras Infirmary, and home sister at the Holborn Union Infirmary.

PRINCESS PATRICIA AUXILIARY HOSPITAL, BRAY, CO. WICKLOW.—Miss MacMahon has been appointed assistant matron. She was trained at Mercer's Hospital, Dublin, where she has since been assistant matron.

BETHNAL GREEN MILITARY HOSPITAL.—Miss D. M. E. Riggs has been appointed ward sister. She was trained at the Royal Berkshire Hospital, Reading, where she was later successively sister in charge of the out-patient department, the ophthalmic wards, and the theatre.

(*Continued on page* 464.)

### Matrons' and Sisters' Appointments—(*cont.*).

Bethnal Green Infirmary, N.E.—Miss Florence Mary Foley has been appointed sister. She was trained at the Mile End Infirmary and at the North-Eastern Fever Hospital, and was later staff nurse at the former institution.

Brynkal Auxiliary Hospital, Chirk, Denbighshire.—Sister A. E. Griffiths has been appointed sister-in-charge. She was trained at Birkenhead Infirmary, where she was later home sister.

General Hospital, Peru, South America.—Miss Sarah Macdougall has been appointed sister in charge. She was trained at Leith General Hospital, where she later became ward sister, and at Edinburgh City Hospital.

Holborn Union Infirmary.—Miss Ethel Butcher has been appointed home sister, having previously been ward sister and temporary night sister. Miss Butcher was trained at Woolwich Union Infirmary.

Margate, Royal Sea-Bathing Hospital.—Miss E. Hunt has been appointed sister. She was trained at Sheffield Royal Infirmary, and has since done private nursing. She is a member of Queen Alexandra's Royal Naval Nursing Service Reserve.

Paddington Infirmary.—Miss Ethel Johnson has been appointed night sister. She was trained at Holborn Union Infirmary, and has since been ward sister at that institution, and also at Bradford Union Infirmary and at Paddington Infirmary.

Shoreditch Infirmary.—Miss Lilian Curran-Tyrell has been appointed sister. She was trained at Shoreditch Infirmary, where she was later ward sister. She has since done military nursing in Weymouth.

**NOTE.—Particulars of Appointments for insertion in this column will be welcomed.**

Rates of Subscription: Twelve months, 6/6; Six months, 4/-; Three months, 2/-; post free to any address in the United Kingdom. Abroad, Twelve months, 8/8; Six months, 5/-; Three months, 2/6, post free. The Hospital "Index" to Volume LX., April to September 1916, is now ready, price 2½d. per copy, post free. Address The Manager, "The Hospital," The Hospital Building, 28/29 Southampton Street, Strand, London, W.C.

